# Experiences of Patients With a Diabetes Self-Care App Developed Based on the Information-Motivation-Behavioral Skills Model: Before-and-After Study

**DOI:** 10.2196/11590

**Published:** 2019-04-18

**Authors:** Eunjoo Jeon, Hyeoun-Ae Park

**Affiliations:** 1 College of Nursing Seoul National University Seoul Republic of Korea

**Keywords:** diabetes mellitus, self-management, blood glucose self-monitoring, mobile applications

## Abstract

**Background:**

Mobile phones have been actively used in various ways for diabetes self-care. Mobile phone apps can manage lifestyle factors such as diet, exercise, and medication without time or place restrictions. A systematic review has found these apps to be effective in reducing blood glucose. However, the existing apps were developed and evaluated without a theoretical framework to explain the process of changes in diabetes self-care behaviors.

**Objective:**

This study aimed to evaluate the diabetes self-care app that we developed by measuring differences in diabetes self-care factors between before and after using the app with the Information-Motivation-Behavioral skills model of Diabetes Self-Care (IMB-DSC).

**Methods:**

We conducted a single-group pre- and postintervention study with a convenience sample of diabetes patients. A total of 38 adult patients with diabetes who had an Android smartphone were recruited. After conducting a preliminary survey of those who agreed to participate in the study, we provided them with a manual and a tutorial video about the diabetes self-care app. The app has functions for education, recommendations, writing a diary, recording, goal setting, sharing, communication, feedback, and interfacing with a glucometer, and it was applied for 4 weeks. We measured the general characteristics of participants, their history of diabetes self-care app usage, IMB-DSC factors, and blood glucose levels. The IMB-DSC factors of information, personal motivation, social motivation, behavioral skills, and behaviors were measured using an assessment tool consisting of 87 items extracted from the Diabetes Knowledge Test, third version of the Diabetes Attitude Scale, Diabetes Family Behavior Checklist, and Diabetes Self-Management Assessment Report Tool.

**Results:**

The mean age of the participants was 43.87 years. A total 30 participants out of 38 (79%) had type 2 diabetes and 8 participants (21%) had type 1 diabetes. The most frequently used app function was recording, which was used by 34 participants out of 38 (89%). Diabetes self-care behaviors (*P*=.02) and diabetes self-care social motivation (*P*=.05) differed significantly between pre- and postintervention, but there was no significant difference in diabetes self-care information (*P*=.85), diabetes self-care personal motivation (*P*=.57), or diabetes self-care behavioral skills (*P*=.89) between before and after using the diabetes self-care app.

**Conclusions:**

Diabetes self-care social motivation was significantly improved with our diabetes self-care app by sharing experiences and sympathizing with other diabetes patients. Diabetes self-care behavior was also significantly improved with the diabetes self-care app by providing an interface with a glucometer that removes the effort of manual input. Diabetes self-care information, diabetes self-care personal motivation, and diabetes self-care behavioral skills were not significantly improved. However, they will be improved with additional offline interventions such as reflective listening and simulation.

## Introduction

### Background

Diabetes mellitus is a chronic disease caused by an absolute or relative deficiency of insulin [1]. The number of patients with diabetes is increasing worldwide. According to the World Health Organization, 1.5 million deaths were directly caused by diabetes in 2014, and 422 million adults were living with diabetes [2]. The prevalence of diabetes in Korea was 13.7% in 2014, up from 8.6% in 2001 [1]. Diabetes can cause death because of inactivity of the heart, kidneys, eyes, and blood vessels if the blood glucose level is not properly controlled [3].

Antidiabetic medication such as an oral hypoglycemic agent plus insulin is used as an active regimen for blood glucose control to prevent diabetes and diabetes complications; however, drug therapy alone is not sufficiently effective [4]. Diabetes can be effectively treated when drug therapy is combined with appropriate diabetes self-care of lifestyle factors such as diet, exercise, and self-monitoring of blood glucose (SMBG) [5].

Mobile phones have recently been actively used for changing diabetes self-care behaviors. According to a health report from Intercontinental Marketing Services in 2015, diabetes self-care mobile phone apps were the second most common type of chronic disease management apps [6]. Diabetes self-care apps can provide various functions as listed below [6,7]:

1. Providing information in various formats such as text, images, and videos.
2. Allowing users to record their diabetes self-care behaviors.
3. Displaying diabetes self-care behaviors in charts and diagrams.
4. Providing tailored recommendations based on the history of diabetes self-care behaviors entered by users.
5. Providing reminders to perform diabetes self-care.
6. Enabling real-time communication between the medical provider and patient.

Diabetes self-care apps that manage lifestyle factors such as diet, exercise, and medication adherence have been shown to be effective in reducing the blood glucose level. A systematic review and meta-analysis of diabetes self-care apps found that they had a significant improvement in glycemic control [8,9].

However, existing diabetes self-care apps were developed without a theoretical framework to explain the process of changes in diabetes self-care behaviors [10]. Even though there are apps providing evidence-based interventions, they are not tailored to the level of knowledge and status of the diabetes self-care behaviors of the patients [11]. There are only a few apps reflecting user requirements [12] such as providing reminders, utilizing social media, and having an interface with a glucometer for facilitating diabetes self-care [13].

This study developed a diabetes self-care app based on the information-motivation-behavioral skills (IMB) model, which is a behavioral change theory for explaining the process of changing diabetes self-care behaviors and user requirements determined through focus group interviews. The app provides evidence-based tailored information, provides reminders, utilizes social media, and has an interface with a glucometer [14]. The American Association of Diabetes Educators (AADE) recommended evaluating changes in diabetes self-care using the IMB model [15]. On the basis of the AADE’s recommendation, the developed app implemented an information factor to evaluate diabetes education, a motivational factor to evaluate cognitive changes, and a behavioral skills factor to evaluate the self-efficacy of diabetes self-care behaviors [16].

### Objectives

This study evaluated the diabetes self-care app by measuring knowledge about diabetes and diabetes self-care as an information factor, individual and social motivation as a motivational factor, diabetes self-care skills as behavioral skills, and blood glucose levels as the outcomes.

## Methods

### Participants and Procedures

We conducted a single-group pre- and postintervention study with a convenience sample of diabetes patients to evaluate the diabetes self-care app developed in this study [14]. Participants were recruited by applying the following inclusion criteria: being older than 19 years, diagnosed with diabetes, and owning an Android phone. We recruited patients with diabetes regardless of diabetes types based on the previous intervention studies [15,16]. The sample size was 38, which exceeded the required sample size of 34 estimated for a 2-sided significance level of .05, a statistical power of .80, and an effect size for the intervention of .5 [8].

**Figure 1:**
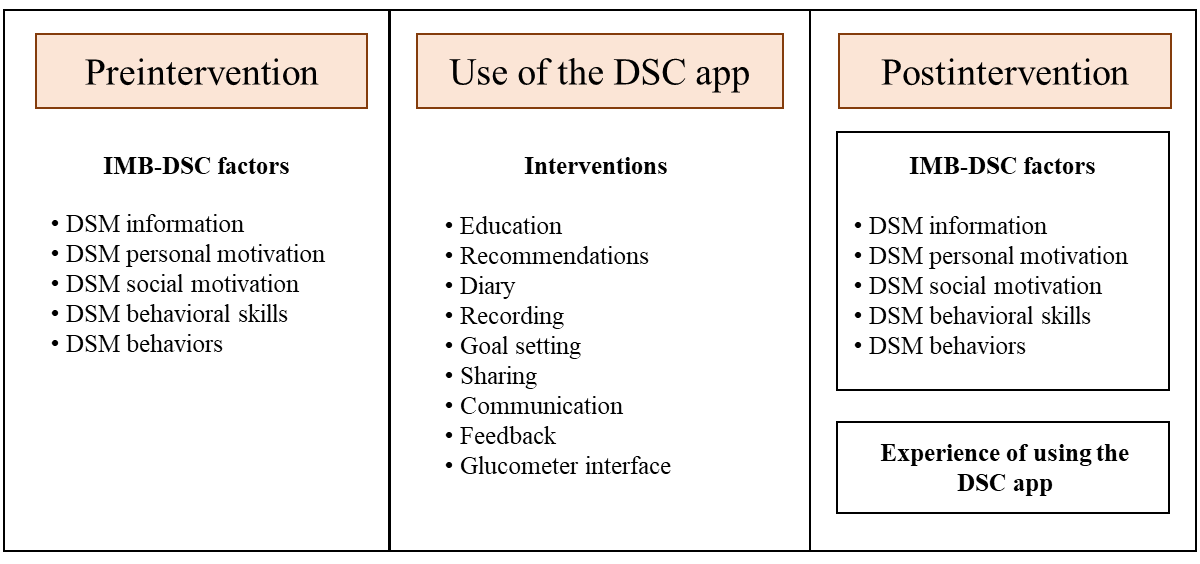
Research process applied in this study. DSM: diabetes self-management; IMB-DSC: Information-Motivation-Behavioral skills model of Diabetes Self-Care.

The research process consisted of the following steps (Figure 1):

1. We recruited study participants by posting a call for participation on 5 self-help websites for patients with diabetes from August 1 to 17, 2016.
2. We surveyed patients with diabetes who agreed to participate using a questionnaire to obtain information about their personal motivation, social motivation, behavioral skills, and behaviors.
3. We provided the subjects with a manual and tutorial video on how to use the diabetes self-care app.
4. The diabetes self-care app applied for 4 weeks provides educational material with recommendations about writing a diary, recording, goal setting, sharing, communication, feedback, and interfacing with the glucometer.
5. We surveyed the IMB-DSC factors after the 4-week intervention using the same questionnaire used in the first step.
6. We used an open-ended questionnaire to survey the subjects’ experiences with using the diabetes self-care app and identify how functions of the app influenced the changes in IMB-DSC factors.

This study was approved by the institutional review boards at the Seoul National University (approval # 1510/002-009).

### Theoretical Framework

**Figure 2:**
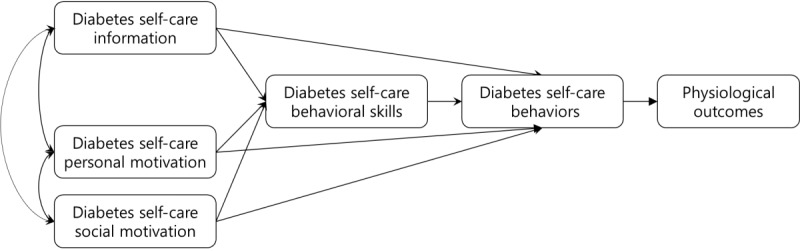
The modified Information-Motivation-Behavioral skills model of Diabetes Self-Care.

The theoretical framework used in this study is presented below (Figure 2). The app was constructed by adding an index for the physiological outcomes to the IMB-DSC proposed by Osborn [17] adapted from the IMB model of Fisher et al [18]. It consists of 6 factors: information, personal motivation, social motivation, behavioral skills, behaviors, and physiological outcomes. Information, personal motivation, and social motivation are correlated with each other and affect behavioral skills and behaviors. Diabetes self-care behavioral skills affect diabetes self-care behaviors, whereas diabetes self-care behaviors affect blood glucose levels as the physiological outcomes.

### Intervention

The app used in this study for intervention was developed by the authors, and the technical aspects such as its algorithm, heuristic, and usability were tested [14]. Our diabetes self-care app developed in the previous work was designed to be user-centered through focus group interviews, provided evidence-based tailored interventions with knowledge extracted from clinical practice guidelines, and integrated diverse functions such as education, recommendations, goal setting, recording, diary writing, social networking, feedback, reminders, and interface with a glucometer. We pilot tested the diabetes self-care app for usability with 14 diabetes patients.

**Figure 3:**
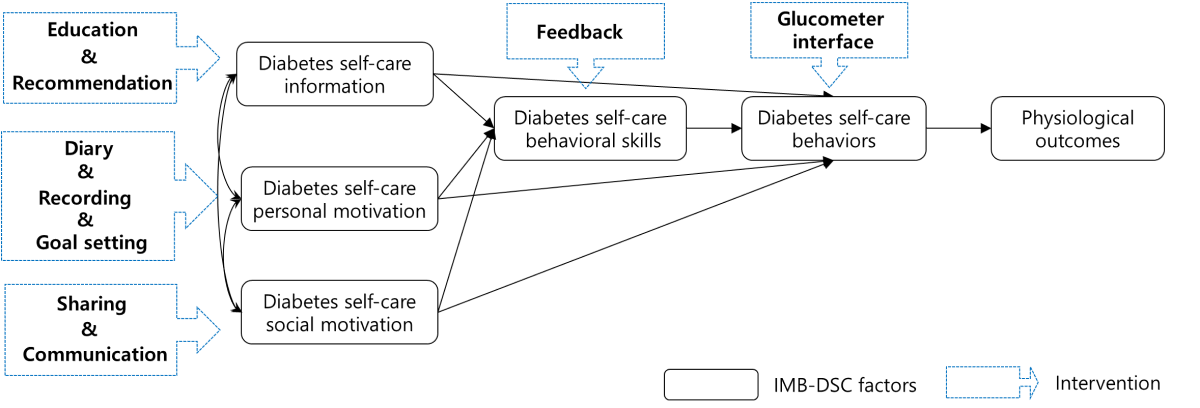
Relationships between the research framework and the diabetes self-care app intervention. IMB-DSC: Information-Motivation-Behavioral skills model of Diabetes Self-Care.

Figure 3 shows how the diabetes self-care app functions are related to the components of the theoretical framework (Figure 3):

1. Evidence-based education and personalized recommendations were included to facilitate the provision of information.
2. Self-reflective diary writing, recording diabetes self-care behaviors, and individual goal setting were included to increase personal motivation.
3. Sharing with other patients and communication with health care providers were included to increase social motivation.
4. Feedback and visualizing blood glucose trends were included to improve behavioral skills.
5. Finally, a wireless glucometer interface (via Bluetooth) for receiving blood glucose data automatically was included to promote diabetes self-care behaviors.

### Measurements and Outcomes

We measured the general characteristics of the study participants, their history of diabetes self-care app usage, IMB-DSC factors, blood glucose levels, and surveyed their experiences with using the diabetes self-care app. We pilot tested the readability of the tools measuring 5 IMB-DSC factors with 14 diabetes patients [14].

#### General Characteristics and History of Using a Diabetes Self-Care App

Demographic and clinical information including age, gender, type of diabetes, and duration of diabetes were collected in the beginning of the study using a survey. The number of diabetes self-care app functions used was collected at the end of the study. The diabetes self-care app includes functions for recording diabetes self-care behaviors (diet, exercise, blood glucose level, medication adherence, blood pressure, and weight), providing reminders, glucometer interfacing, diary writing, and sending text messages to the health care provider.

#### Information-Motivation-Behavioral Skills Model of Diabetes Self-Care Factors and Physiological Outcomes

Diabetes self-care information was measured using the Diabetes Knowledge Test (DKT), which consists of 14 items measuring the general knowledge about diabetes and diabetes self-care [19]. The reliability of the DKT was measured by a Cronbach alpha of .67 in this study and of .71 in the study by Choi [20].

Diabetes self-care personal motivation was measured using the third version of the Diabetes Attitude Scale (DAS-3). The DAS-3 measures blood glucose control (3 items) and patient autonomy (8 items) using a 5-point scale ranging from 1 (strongly disagree) to 5 (strongly agree) [21]. The reliability of blood glucose control and patient autonomy in DAS-3 was measured by Cronbach alpha values of .57 and .71, respectively, in this study and .63 and .72, respectively, in the study by Choi [20].

Diabetes self-care social motivation was measured using the Diabetes Family Behavior Checklist (DFBC). The DFBC consists of 5 items with a 5-point scale ranging from 1 (“not at all”) to 5 (“at least once a day”) to measure social support in diabetes self-care [22]. The reliability of DFBC was measured by a Cronbach alpha of .84 in this study and of .87 in the study by Chang and Song [23].

Diabetes self-care behavioral skills were measured using the behavioral skills assessment tool that is part of the Diabetes Self-Management Assessment Report Tool (D-SMART). The tool consists of 20 items in 6 subcategories (diet, exercise, SMBG, medication, reducing risk factors, and problem solving) that are scored on a 4-point scale ranging from 1 (“I can’t do it”) to 4 (“I can do it for sure”) [24]. The reliability of the behavioral skills assessment tool of D-SMART was measured by a Cronbach alpha of .89 in this study and of .62 in the study by Choi [20].

Diabetes self-care behaviors were measured using the behaviors assessment tool of the D-SMART, which consists of 39 items in the 6 subcategories used to measure diabetes self-care behavioral skills [24]. The reliability of that behavior’s assessment tool was measured by a Pearson correlation coefficient of .79 in this study and of .73 in the study by Choi [20].

The physiological outcomes of the preprandial and postprandial blood glucose levels were measured by a glucometer.

#### Experiences With Using the Diabetes Self-Care App

We used an open-ended questionnaire to survey the experiences of the subjects with using the diabetes self-care app during the 4-week study period. The questionnaire asked whether IMB-DSC factors had improved, and if they had, which aspects of the app had contributed to these improvements.

### Data Analysis

The collected data were analyzed using SPSS (version 22.0; SPSS Korea). The general characteristics of the subjects and the usage history of the diabetes self-care app were analyzed with descriptive statistics such as the mean and SD. Whether the IMB-DSC factors and the blood glucose level conformed to a normal distribution was tested using the Shapiro-Wilk test. Differences in IMB-DSC factors and the blood glucose level between pre and post intervention were analyzed using paired *t* tests when normality was satisfied and using Wilcoxon signed-rank tests in the other cases. We also analyzed the differences in IMB-DSC factors by gender, age (below average and above), diabetes type, and length of the condition (under 1 year and other). The normality in subgroups was tested, and a Wilcoxon singed-rank test was performed when normality was violated. Finally, the directed dependencies among the IMB-DSC factors were tested using the path analysis with postintervention values.

## Results

### General Characteristics of the Study Subjects

Demographic and clinical characteristics of the participants are presented (Table 1). More than half of them were male, their mean age was 43.87 years, and the largest proportion of them were in their 40s (n=18, 47%), followed by their 30s (n=11, 29%). Of the 38 participants, 30 (79%) had type 2 diabetes and 8 (21%) had type 1 diabetes. The mean duration of diabetes was 16.25 years in type 1 diabetes and 6.26 years in type 2 diabetes.

### Usage History of the Diabetes Self-Care App

The diabetes self-care app was accessed an average of 21.79 times during the 4-week study period, ranging from 11 to 33. The most frequently used function was the recording function, with 34 out of 38 subjects (89%) using it. The next most frequently used functions were providing reminders and the glucometer interface, with 22 out of 38 subjects (58%) using these functions (Table 2).

### Differences in Information-Motivation-Behavioral Skills Model of Diabetes Self-Care Factors

The preintervention scores, postintervention scores, and results of tests of the changes in IMB-DSC factors are presented in Table 3. Diabetes self-care social motivation differed significantly before (mean 12.16 [SD 5.48]) and after intervention (mean 13.87 [SD 6.81]; *P*=.05), as did diabetes self-care behaviors (mean 40.84 [SD 7.30] and mean 42.58 [SD 5.92], respectively; *P*=.02). However, there was no significant difference in information (*P*=.85), personal motivation (*P*=.57), or behavioral skills (*P*=.89) between before and after use of the diabetes self-care app.

**Table 1:** Demographic and clinical characteristics of the participants (N=38).

Characteristics		Participants
**Gender, n (%)**		
	Male	23 (61)
	Female	15 (39)
**Age (years), n (%)**		
	20-29	1 (3)
	30-39	11(29)
	40-49	18 (47)
	50-59	6 (16)
	60-69	2 (5)
**Type of diabetes, n (%)**		
	Type 1	8 (21)
	Type 2	30 (79)
**Duration of diabetes (years), mean (SD)**		
	Type 1	16.25 (14.83)
	Type 2	6.26 (7.27)

**Table 2:** Numbers of subjects using the various diabetes self-care app functions (N=38).

Function	Total uses, n	Subjects, n (%)
Recording	3118	34 (89)
Reminders	55	22 (58)
Glucometer interface	1892	22 (58)
Sharing	56	10 (26)
Communication	10	10 (26)

**Table 3:** Comparison of independent variables between pre- and postintervention (N=38).

Variable		Maximum, n	Preintervention, mean (SD)	Postintervention, mean (SD)	*t* or *Z* value	*P* value
Diabetes self-care information		14	10.92 (1.78)	10.87 (2.37)	–.19	.85
Diabetes self-care personal motivation		45	33.29 (3.30)	33.36 (2.79)	–.57^a^	.57
Diabetes self-care social motivation		25	12.16 (5.48)	13.87 (6.81)	2.06	.05^b^
Diabetes self-care behavioral skills		72	54.89 (8.10)	54.08 (7.55)	–.14^a^	.89
**Diabetes self-care behaviors**						
	Total	73	40.84 (7.30)	42.58 (5.92)	2.54	.02^b^
	Diet	15	9.34 (2.08)	9.87 (2.27)	–1.32^a^	.17
	Exercise	12	7.68 (3.20)	7.63 (2.79)	–.59^a^	.56
	Self-monitoring of blood glucose	7	5.34 (2.23)	5.92 (1.44)	–1.93^a^	.05^b^
	Problem solving	20	5.55 (2.91)	6.11 (3.09)	–2.07	.04^b^
	Reducing risk factors	19	12.92 (3.23)	13.05 (2.85)	0.38	.71

^a^Wilcoxon signed-rank test done.

^b^*P*<.05.

### Differences in Information-Motivation-Behavioral Skills Model of Diabetes Self-Care Factors by General Characteristics

There were no significant differences in changes of information, personal motivation, and behavioral skills before and after intervention by general characteristics such as sex and age. However, there were significant differences in changes of social motivation and behavior before and after intervention by type of diabetes and length of condition. In detail, diabetes self-care social motivation in the type 1 diabetes group differed significantly before (mean 11.38 [SD 5.37]) and after the intervention (mean 14.75 [SD 5.52]; *P*=.05), as in length of condition less than the 1-year group differed significantly before (mean 11.40 [SD 4.54]) and after the intervention (mean 13.36 [SD 6.02]; *P*=.03; Table 4).

Diabetes self-care behavior in the type 2 diabetes group differed significantly before (mean 40.87 [SD 7.89]) and after the intervention (mean 42.89 [SD 6.41]; *P*=.02), as in length of condition over the 1-year group differed significantly before (mean 38.15 [SD 8.28]) and after the intervention (mean 41.54 [SD 6.50]; *P*=.02; Table 5).

### Diabetes-Related Physiological Outcomes

The preprandial blood glucose level was not different significantly before and after the intervention using the diabetes self-care app (*P*=.67), but there was a trend in decreasing the postprandial blood glucose level (mean 162.65 [SD 52.91] and mean 137.29 [SD 34.63] mg/dL, respectively; *P*=.09; Table 6).

**Table 4:** Differences in social motivation factor by general characteristics (N=38).

Variable		Samples, n	Preintervention, mean (SD)	Postintervention, mean (SD)	*t* or *Z* value	*P* value
**Type of diabetes**						
	Type 1	8	11.38 (5.37)	14.75 (5.52)	–1.90^a^	.05^b^
	Type 2	30	12.37 (5.44)	13.63 (7.18)	1.28	.21
**Duration of diabetes**						
	Under 1 year	13	13.62 (6.92)	14.85 (8.31)	.67	.51
	Over 1 year	25	11.40 (4.54)	13.36 (6.02)	2.29	.03^b^

^a^Wilcoxon signed-rank test done.

^b^*P*<.05.

**Table 5:** Differences in diabetes self-care behavior factor by general characteristics (N=38).

Variable		Samples, n	Preintervention, mean (SD)	Postintervention, mean (SD)	*t* or *Z* value	*P* value
**Type of diabetes**						
	Type 1	8	40.63 (4.81)	41.00 (2.93)	–.34^a^	.73
	Type 2	30	40.87 (7.89)	42.89 (6.41)	2.58	.02^b^
**Duration of diabetes**						
	Under 1 year	13	38.15 (8.28)	41.54 (6.50)	2.91	.02^b^
	Over 1 year	25	42.20 (6.46)	43.04 (5.58)	1.03	.31

^a^Wilcoxon signed-rank test done.

^b^*P*<.05.

**Table 6:** Comparison of blood glucose levels between pre and postintervention.

Time	Preintervention (mg/dL), mean (SD)	Postintervention (mg/dL), mean (SD)	*t* or *Z* value	*P* value
Preprandial (n=8)	125.70 (53.99)	129.14 (34.56)	–.42^a^	.67
Postprandial (n=18)	162.65 (52.91)	137.29 (34.63)	–1.79	.09

^a^Wilcoxon signed-rank test done.

**Figure 4:**
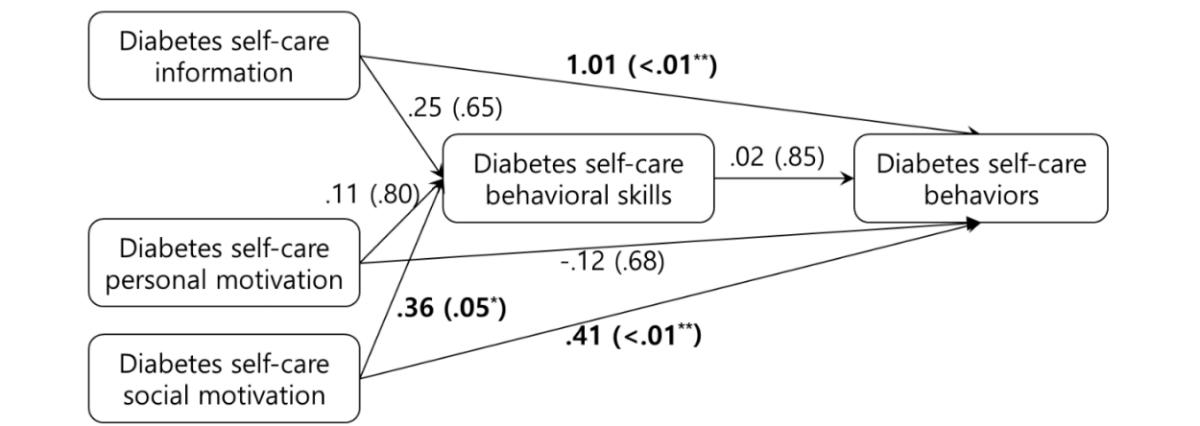
The result of path analysis for Information-Motivation-Behavioral skills model of Diabetes Self-Care. Asterisk signifies significance at *P*<.05 and double asterisk signifies significance at *P*<.01.

### Path Analysis of Information-Motivation-Behavioral Skills Model of Diabetes Self-Care

Diabetes self-care behavior was significantly affected by information (coefficient=1.01; *P*=.008) and social motivation (coefficient=.41; *P*=.002), and diabetes self-care behavior skills were significantly affected by social motivation (coefficient=.36; *P*=.05; Figure 4).

### Experiences With Using the Diabetes Self-Care App

The narratives of the 28 participants who responded to the open-ended questionnaire about their experiences with using the diabetes self-care app are presented (Table 7).

**Table 7:** Comments from the survey of experiences with using the diabetes self-care app (N=28).

Factor	Comments
Diabetes self-care information	“The diabetes self-care app provided general information about diabetes care.”
	“The information provided by the diabetes self-care app may be helpful to the early onset diabetic.”
	“More specific information is needed.”
Diabetes self-care personal motivation	“I am managing myself before using the diabetes self-care app, so I am not sure about the change.”
	“Seeing my records of diet and exercise is motivating me.”
	“Through the diabetes self-care app, the goal of blood glucose control was re-recognized.”
	“I was more interested because I had to check daily in the diabetes self-care app about foot care.”
Diabetes self-care social motivation	“With the diabetes self-care app, I felt like sharing similar experiences.”
	“I liked the ‘Like’ function.”
	“I was stimulated by watching other people’s blood sugar on the bulletin board.”
Diabetes self-care behavioral skills	“The diabetes self-care app has helped me realize that it is difficult to lower an elevated blood glucose level.”
Diabetes self-care behaviors	“The glucometer interface made it possible to conduct blood glucose tests at both work and home.”
	“It is convenient to input the blood glucose value via the glucometer interface.”

## Discussion

### Overview

The purpose of this study was to improve the factors of IMB-DSC, which was validated from previous papers [17,18,25]. As a result, the diabetes self-care app developed in this study was effective in promoting diabetes self-care behaviors and diabetes self-care social motivation and in reducing the blood glucose level. However, it did not have a significant effect on diabetes self-care behavioral skills, diabetes self-care information, or diabetes self-care personal motivation. In addition, the causal relationship of factors of the IMB-DSC was analyzed by path analysis with postintervention measurements. As the number of subjects in this study was small, it was not possible to perform a structural equation modeling (SEM) analysis which can handle measurement error and indirect effect.

### Diabetes Self-Care Information

Diabetes self-care information did not change significantly between before and after use of the app. Our study subjects had high initial scores for diabetes self-care information, so there was not much room to improve after using the app. The initial diabetes self-care information score was much higher in this study than that by Choi, which found significant changes between before and after use of an app [20]. This difference in the initial diabetes self-care information scores between the 2 studies could have been because of the present subjects being younger, making them better able to acquire information using their mobile phones compared with the participants in the study by Choi.

Another possible reason for the absence of a significant increase in the diabetes self-care information could be the large variance in the duration of diabetes. The survey of experiences with using the diabetes self-care app revealed that the participants who had a long history of diabetes considered the app to be more useful for people with early-stage diabetes than for long-term patients. It, therefore, appears to be necessary to provide different sets of diabetes self-care information tailored to patients at different stages. For example, more general information about diabetes and diabetes complications could be provided to early-stage patients and more advanced and specific information about diabetes self-care behaviors could be provided to long-term patients [20,25].

### Diabetes Self-Care Personal Motivation

Diabetes self-care personal motivation did not change significantly between before and after use of the app. This could have been due to the intervention period of 4 weeks being too short to change personal motivation. Previous studies have suggested that long-term interventions lasting more than 6 months are needed to change diabetes self-care personal motivation [25]. Besides, it is difficult to change personal motivation by an online intervention such as writing a diary, recording, and setting a goal alone. A previous study suggested using face-to-face consultation such as reflective listening [17].

### Diabetes Self-Care Social Motivation

Diabetes self-care social motivation differed significantly between before and after use of the app. We introduced a bulletin board where the participants could use to share their diabetes self-care experiences and express empathy with other participants, with the aim of promoting social motivation. Increase in social motivation with using the bulletin board is consistent with the findings of previous studies in which the participants shared their know-how about diabetes self-care and feelings with other patients with the same disease [25-27]. This finding was also confirmed in the open-ended diabetes self-care app experience survey in which study participants expressed that they felt like sharing similar experiences using the bulletin board.

### Diabetes Self-Care Behavioral Skills

Diabetes self-care behavioral skills did not differ significantly between before and after use of the app. Previous studies have recommended the need to demonstrate skills to promote behavioral skills [20,28]. However, we did not provide face-to-face education to increase diabetes self-care behavioral skills; instead, information on diabetes self-care was provided on an app. We suggest providing diabetic patients with information about diet and exercise planning as well as blood glucose measurement scheduling using the simulation technique proposed by Osborn [17].

Diabetes self-care behavioral skills was not significantly affected by diabetes self-care information, which is similar to the findings of Choi [20]. Diabetes self-care behavioral skills were not significantly affected by personal motivation either. However, diabetes self-care social motivation significantly affected diabetes self-care behavioral skills (coefficient=.36; *P*=.05). These findings were different from those of the previous study [20]. However, it is difficult to directly compare the results of this study with those of the previous study as Choi combined personal motivation and social motivation into a single motivation metric [20].

### Diabetes Self-Care Behaviors

There were significant changes in diabetes self-care behaviors between before and after use of the app, especially in SMBG and problem solving. We provided a wireless glucometer interface to receive data on the blood glucose level automatically to minimize the amount of manual input required, which is in accordance with the suggestion of Intercontinental Marketing Services to improve SMBG [6]. A wireless glucometer interface function could have improved SMBG in this study.

Our path analysis showed that information (coefficient=1.01; *P*=.008) and social motivation (coefficient=.41; *P*=.002) significantly affected diabetes self-care behaviors in this study. The effect of social motivation on diabetes self-care behaviors was consistent with the findings of previous studies [25,29]. Increasing social motivation will contribute to increasing diabetes self-care behaviors, but the effect of diabetes self-care information on diabetes self-care behaviors varied in previous studies [20,25,30]. These discrepancies could have been because of diabetic knowledge measurement tools often requiring more general knowledge on diabetes or diabetic complications than the knowledge required to perform diabetes self-care behaviors [20].

Diabetes self-care personal motivation did not significantly affect diabetes self-care behaviors in this study, which contrasts with the findings of Osborn and Egede [28]. This could be because of a difference in how the motivation of the IMB model was measured: Osborn and Egede measured diabetes self-care personal motivation based on diabetes fatigue [28], whereas it was measured based on the attitude to diabetes in this study.

Diabetes self-care behavioral skills did not significantly affect diabetes self-care behaviors in this study. This directly contradicts the finding of Choi [20], which is the only other study that has measured behavioral skills in the same way as in this study.

### Physiological Outcomes

There was a trend toward significant changes in physiological outcomes before and after using the app, especially in the postprandial blood glucose level. This is consistent with the systematic review of Liang et al finding that a diabetes self-care app was effective at improving glycemic control [8,9]. The diabetes self-care app developed in this study appears to reduce the blood glucose level by improving diabetes self-care behaviors. According to the AADE, improvements in diabetes self-care behaviors such as diet, exercise, SMBG, problem solving, and reducing risk factors are effective for blood glucose control [5].

The limitations of this study are as follows: First, it was developed based on Android and so excluded other mobile phone operating systems such as iOS, Windows, and Blackberry OS. Second, although there are some studies that performed a short intervention for 4 to 6 weeks to improve diabetes self-management [16,31,32], the long-term effect of diabetes self-care app cannot be confirmed in this study. Therefore, a long-term study of at least 3 to 6 months is necessary to evaluate the change of glycated hemoglobin reflecting the blood glucose level over the previous 3 months and the personal motivation factor representing belief and attitude about health. Finally, a structural equation model could not be established to explain the effect of the functions provided when using the diabetes self-care app because of the small number of study subjects. A future study needs to conduct an SEM analysis to identify the relationships of IMB-DSC factors with the diabetes self-care app.

### Conclusions

We have developed a diabetes self-care app based on the IMB model that provides personalized education and information to promote diabetes self-care information according to the knowledge level and behavior status of patients, a bulletin board that allows patients to communicate and sympathize with other diabetes patients, and an interface with a glucometer to improve diabetes self-care behavior factors. This study found that the intervention did not produce any significant changes in personal motivation or behavioral skills; these factors could be improved by providing face-to-face counseling or practicing strategies in combination with using the diabetes self-care app.

## Abbreviations

AADE: American Association of Diabetes Educators
DAS-3: Diabetes Attitude Scale
DFBC: Diabetes Family Behavior Checklist
DKT: Diabetes Knowledge Test
D-SMART: Diabetes Self-Management Assessment Report Tool
IMB-DSC: Information-Motivation-Behavioral skills model of Diabetes Self-Care
SEM: structural equation modeling
SMBG: self-monitoring of blood glucose
